# Abductor pollicis longus tendon interposition for arthrosis of the first carpo-metacarpal joint. Long-term results

**DOI:** 10.1186/s12891-016-0910-5

**Published:** 2016-02-01

**Authors:** Line Lied, Kari Bjørnstad, Ann K. N. Woje, Vilhjalmur Finsen

**Affiliations:** Department of Orthopaedic Surgery, St. Olav’s University Hospital, 7006 Trondheim, Norway; Department of Clinical Services, St. Olav’s University Hospital, Trondheim, Norway; Department of Neuroscience, NTNU, Trondheim, Norway

**Keywords:** Basal joint arthrosis, Interposition plasty, Abductor pollicis longus, Thumb, Arthritis, Long-term results

## Abstract

**Background:**

We performed an interposition arthroplasty using the abductor pollicis longus tendon for arthrosis in the basal joint of the thumb that needed surgery from 1995 to 2010. In 2001 47 patients (55 thumbs) were reviewed after 3.5 (1–5) years. The pain relief was excellent in 32 thumbs, and 25 patients improved their ability to perform daily tasks. Mobility was well preserved. Key pinch and grip strengths averaged 78 % and 89 %, respectively, of those in unaffected hands. We have now re-examined all 33 available patients (36 thumbs) 11–14 years after surgery.

**Methods:**

Fourty one of the originally examined patients were still alive. Seven were too ill to attend a follow-up and one refused. The remainder were examined in a fashion as similar as possible to that at the original review. The patients’ subjective estimations of pain during the last week and satisfaction with the cosmetic and general results were recorded on visual analogue scales. The patients’ ability to perform various activities of daily living were recorded and they completed the Disability of the arm, shoulder and hand (DASH) questionnaire. The mobility of the wrist and abduction of the thumb of the operated hands were recorded with a goniometer. Grip and pinch strength were measured and new radiographs were obtained.

**Results:**

Key pinch strength had increased significantly over the last 10 years. The mobility was still good, except for thumb abduction, which had decreased with time. The median DASH score had fallen from 28 to 20 between the two reviews. There was insignificant further median loss of distance between the scaphoid and the metacarpal since the earlier review.

**Conclusions:**

The good results of this procedure found soon after surgery are maintained long-term.

## Background

Several interposition techniques have been described for surgical treatment of arthrosis in the first carpometacarpal joint. Most are reported to give good short and medium term results. However, the long-term results of this type of surgery are less well documented.

During the years 1995 to 2010 we performed an interposition arthroplasty using the abductor pollicis longus (APL) tendon when surgery was required [1]. A total of 47 of the patients operated during the period 1996 to 1999 were re-examined 3.5 years after surgery [2]. We found the results to be satisfactory. The purpose of the present study was to investigate the long-term results in the same patient group.

## Methods

Fifty two patients were operated with an APL interposition arthroplasty in our department during a 4-year period (1996–1999). The entire trapezium was excised through a dorsoradial incision and a distally based strip consisting of the radial 1/3 of the abductor pollicis longus tendon was prepared. This strip was used to twist the flexor carpi radialis and APL tendons together, securing the volar and ulnar aspects of the first metacarpal bone [1]. The hand was immobilized in plaster of Paris for four to five weeks postoperatively.

**Fig. 1:**
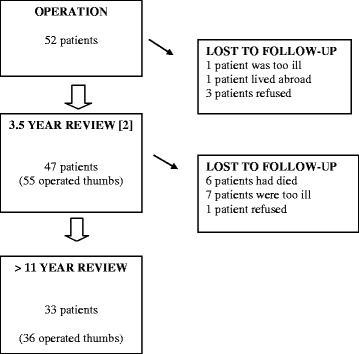
Flow chart of patients operated during 1996–1999

In 2001 we re-examined 47 of these patients with 55 operated thumbs after a mean follow-up of 3.5 years (16–60 months) [2]. Forty-one of the patients examined in 2001 were still alive in 2011. Seven could not attend a second follow-up because of illness or advanced age and one refused to participate (Fig. 1). The remaining 33 patients (29 women and 4 men), with 36 operated thumbs were re-examined a mean of 13 (11–14) years after surgery and the state of their operated hands compared to the findings at the 3.5 year review. These patients were 56 (range: 44–66) years old at the time of surgery and 69 (55–80) years old at review. By the time of this last review 19 patients had been operated for carpometacarpal arthrosis also in the contralateral hand. Only the hands operated during the original study period were investigated in the present follow-up.

In the present review two hand therapists (KB and AKNW) repeated the examination of the previous follow-up as far as possible. The patients’ subjective estimations of pain during the last week and satisfaction with the cosmetic and general results were recorded on visual analogue scales. These recordings were converted to scores (VAS) ranging from 0 (best) to 100 (worst imaginable). VAS scores of 15 or lower were, as in the previous review, arbitrarily taken to indicate an excellent outcome. The patients were furthermore interviewed regarding their ability to perform various activities of daily living before the operation and at the two follow-ups (Table 1) and completed the Disability of the arm, shoulder and hand (DASH) questionnaire [3, 4]. In addition, the patients completed the Patient rated wrist and hand evaluation (PRWE) questionnaire in the present review [5, 6]. Both questionnaires are validated in Norwegian [4, 7]. The mobility of the wrist and abduction of the thumb of the operated hands were recorded with a goniometer. Grip and pinch strength were measured with a Jamar dynamometer (J.A. Preston Corp., Clifton, NJ, USA). The median value of three attempts was noted. No corrections were made for non-dominant hands. Fifteen patients had been arbitrarily chosen for a radiographic examination at the review in 2001. New radiographs were obtained in all the 12 (15 reviewed hands) of these 15 patients who attended the present review. In order to compare with the 3.5 years follow-up, the present radiographs were taken without provocation or loading.

**Table 1:** Ability to perform selected activities of daily living before surgery and at 3.5 [2] and 13-year review

	Pre-op		3.5 year review [2]			13 year review		
	*n* = 55		*n* =55			*N* =36		
	Yes	No	Same	Better	Worse	Same	Better	Worse
Open a car door	42	13	42	11	2	18	17 *	1
Use a doorkey	45	10	47	7	2	16	20 **	0
Open a jar of jam	13	42	36	14	5	20	13	3
Wring a cloth	25	30	30	21	4	21	15	0

(Better = Unable before surgery, able at follow-up

Worse = Able before surgery, unable at follow-up)

* *p* < 0.01; ** *p* < 0.001 compared to 3.5 year review

In accordance with Norwegian regulations, most of the data and the key connecting patients with their clinical data were destroyed once the 3.5 year follow-up had been completed. It was therefore not possible to compare each individual patient’s data at the two reviews. We could only compare averages at the two points in time. The exception was the radiographic data, where each patient could be identified.

Fisher’s exact test was used for statistical evaluation of categorical variables and the Student’s *t*-test for continuous variables.

Patients had been contacted by mail with a letter describing the present study and asking them to attend the hospital for this review. It was assumed that those who chose to attend also consented to taking part in the study. No written consent was therefore obtained. The study and this manner of obtaining patient consent were explicitly approved by The Central Norwegian Committee for Medical and Health Research Ethics (Registration number: 2010/1267-2).

## Results

The median VAS scores and the proportions of patients who reported excellent results (VAS < 16) were similar to those found at the 3.5-year review (Table 2).

**Table 2:** Median (range) VAS and DASH scores (0 = best; 100 = worst) and the number with VAS scores that were considered excellent (VAS < 16)

	3.5-year review [2] (*n* = 55)		13-year review (*n* = 36)	
		Excellent		Excellent
Pain (VAS)	11 (0–85)	36 (65 %)	15 (0–75)	22 (61 %)
Cosmesis (VAS)	5 (0–100)	37 (67 %)	8 (0–98)	33 (92 %)
General satisfaction (VAS)	5 (0–100)	34 (62 %)	15 (0–89)	24 (67 %)
Regretted surgery	4 (7 %)		3 (8 %)	
DASH score	28 (0–84)		20 (0–62)	
	(*n* = 47)		(*n* = 33)	

“Regretted surgery”: Those who stated that they would not have consented to surgery if they had known the outcome in advance)

The proportion who reported improvement in opening a car door and in using a door key had increased significantly from the 3.5 year to the present review (Table 1).

The median DASH score had fallen from 28 to 20 between the two reviews (Table 2). The median PRWE score was 22 (0–70). Three of the 33 patients replied that they would not have consented to surgery if they had known the outcome in advance.

Thumb radial abduction was decreased significantly compared to the results from the 3.5 year review, while the wrist mobility was almost unchanged (Table 3). The distal phalanx of the 5^th^ finger could be reached by 34 of the 36 operated thumbs.

**Table 3:** Mean (SD) mobility and strength in the operated hands at 3.5 [2] and 13-year follow-up

	3.5-year review [2] (*n* = 55)	13-year review (*n* = 36)
Thumb radial abduction (°)	51 (16)	36 (9)*
Wrist extension (°)	62 (12)	59 (10)
Wrist flexion (°)	68 (15)	71 (8)
Ulnar deviation (°)	36 (10)	32 (7)
Radial deviation (°)	21 (6)	18 (5)
Key pinch (kg)	3.8 (1.4)	5.0 (1.5)*
Power grip (kg)	21 (10)	24 (6)

*p < 0.001 compared to operated hands at 3.5-year review

There was little difference in grip strength between the 3.5 year and the present review, but the mean pinch strength had increased significantly (Table 3).

The median distance between the scaphoid and the first metacarpal bone on the radiographs was 11 (8–14) mm before surgery, 4.9 (1–11) mm at the 3.5 year review and 4.3 (1–6) mm for the same patient group at the present review.

## Discussion

We have not found many long-term studies of the results of treatment of basal joint arthrosis. Because of the data protection regulations in Norway, it was not possible to compare values for individual patients at the two reviews, except for radiological data. This report must thus be regarded as a long-term review with comparison of results to those in a very similar population after a shorter review. This should, however, still make it possible to decide whether or not the short time gains after this procedure are maintained in the longer term.

The median VAS score for pain in our patients was 15, and 22 thumbs (61 %) had a VAS score < 16. This is not as good as the results of Elfar and Burton [8] who reported 95 % excellent pain relief 11 years after trapeziectomy with ligament reconstruction and tendon interposition. Kaarela and Raatikainene [9] reported of 76 % good/excellent pain relief six years after tendon interposition. These studies had other criteria for excellence than those used in the present study.

The mean DASH score in the present review was 20 compared to 28 at the 3.5 year review. At the time of the earlier review the mean age of the patients was 62 years [2], while it was 69 years at the time of the present review. This increase in mean age would have led one to expect an increase in mean DASH scores at the present review [10]. This was not the case, indicating that there was probably no worsening of DASH scores due to the operated basal joint arthrosis.

Avisar et al. [11] evaluated 13 patients and 15 thumbs 15 years after trapeziectomy with abductor pollicis longus tendon interposition. Mean DASH score was 17, wich is similar to the score in our study. This seems to indicate that tendon interposition plasties give good results also 13–15 years after surgery.

The radiological median distance between the scaphoid and the first metacarpal was 4.3 mm at the present review. This is almost unchanged since the 3.5 year review but less than the 7 mm that Basar et al. [12] reported 6 years after ligament reconstruction and tendon interposition.

Mean grip strength more than 11 years after surgery was slightly better than after 3.5 years, and mean key pinch had increased significantly. The mean grip strength for the women in our study was 23 kg, which matches the normative data for Grip strength for women at the age of 69 years of 18.5–22.5 kg [13]. The normative data for key pinch for women at the age of 69 years is 6.5–6.8 kg [13], while the mean key pinch for the women in our study was somewhat lower (5.0 kg). Both the grip strength and key pinch measurements in our study are similar to other long-term studies [11, 14].

## Conclusions

Although there is some reduction in thumb abduction from 3.5 to 13 years after surgery, other gains after surgery are retained and in some instances slightly improved. We therefore conclude that the improvements obtained in the short term after this procedure are maintained also long-term.

## Abbreviations

DASH: Disabilities of the arm shoulder and hand questionnaire
APL: Abductor pollicis longus tendon - VAS: Visual analogue score
